# Silybin supplementation during HCV therapy with pegylated interferon-α plus ribavirin reduces depression and anxiety and increases work ability

**DOI:** 10.1186/s12888-016-1115-z

**Published:** 2016-11-15

**Authors:** Giulia Malaguarnera, Gaetano Bertino, Giuseppe Chisari, Massimo Motta, Michele Vecchio, Marco Vacante, Filippo Caraci, Carmela Greco, Filippo Drago, Giuseppe Nunnari, Michele Malaguarnera

**Affiliations:** 1 Research Center “The Great Senescence”, University of Catania, Catania, Italy; 2 Department of Biomedical and Biotechnological Sciences, University of Catania, Catania, Italy; 3 Department of Experimental and Clinical Medicine, University of Catania, Catania, Italy; 4 U.O.C Physical Medicine and Rehabilitation, A.O.U. Policlinico Vittorio Emanuele, Catania, Italy; 5 Department of Drug Sciences, University of Catania, Catania, Italy; 6 IRCCS Associazione Oasi Maria S.S., Institute for Research on Mental Retardation and Brain Aging, Troina, Italy

**Keywords:** Silybin, Interferon, Hepatitis C, Depression, Anxiety, Work ability index

## Abstract

**Background:**

Hepatitis C virus infection and interferon treatment are often associated with anxiety, depressive symptoms and poor health-related quality of life.

To evaluate the Silybin-vitamin E-phospholipids complex effect on work ability and whether health related factors (anxiety and depression) were associated with work ability in subjects with chronic hepatitis C treated with Pegylated-Interferon-α2b (Peg–IFN) and Ribavirin (RBV).

**Methods:**

Thirty-one patients (Group A) with chronic hepatitis and other 31 subjects in Group B were recruited in a randomized, prospective, placebo controlled, double blind clinical trial. Group A received 1.5 mg/kg per week of Peg–IFN plus RBV and placebo, while Group B received the same dosage of Peg–IFN plus RBV plus association of Silybin 94 mg + vitamin E 30 mg + phospholipids 194 mg in pills for 12 months. All subjects underwent to laboratory exams and questionnaires to evaluate depression (Beck Depression Inventory - BDI), anxiety (State-trait anxiety inventory - STAI) and work ability (Work ability Index - WAI).

**Results:**

The comparison between group A and group B showed significant differences after 6 months in ALT (*P* < 0.001), and viremia (*P* < 0.05), after 12 months in ALT (*P* < 0.001), and AST (*P* < 0.001), at follow up in AST (*P* < 0.05), and ALT (*P* < 0.001). Significant difference were observed after 1 month in WAI (*p* < 0.001) and BDI (*P* < 0.05), after 6 months in WAI (*P* < 0.05) and STAI (*P* < 0.05), after 12 months and at follow up in WAI, STAI and BDI (*p* < 0.01).

**Conclusions:**

The supplementation with Silybin-vitamin E -phospholipids complex increased work ability and reduced depression and anxiety in patients treated with Peg–IFN and RBV.

**Trial registration:**

NCT01957319, First received: September 25, 2013. Last updated: September 30, 2013 (retrospectively registered).

## Background

Hepatitis C virus (HCV) is the most common cause of cirrhosis and hepatocellular carcinoma in the United States and the Western world [1, 2]. Chronic hepatitis C virus (HCV) infection is a systemic disease leading to hepatic and extra-hepatic manifestations [3–5]. Interferon-α (IFN-α) forms a mainstay of treatment for chronic HCV infection and is usually combined with ribavirin and/or protease inhibitors. IFN-α has antiviral, anti-proliferative and immunomodulatory activities [6]. Both HCV and IFN treatment have shown depression, distress, psychosocial burden and resources, and poor health-related quality of life. Work plays an important role in daily life activities, and is a strong motivator for social status and for social contacts. The interferon produces often in patients with HCV depressive symptoms, myalgia, anhedonia, mood alterations, which may have a negative effect in the ability to perform work, in social functions, and in both physical and mental activities. The impairment in both physical function, such as running and lifting difficulties, and in mental activities, such as fatigue and distress, is not attributable to interferon, but it is also exacerbated by ribavirin [7, 8]. These potentially adaptive behavioral responses to cytokines not only can benefit the chronic exposure to elevate both inflammatory and pro-inflammatory cytokines, but also persistent alterations in neurotransmitter function and behavior can lead to the development neuropsychiatric dysfunction [9]. Our efforts in HCV treatment have been focused on understanding the impact of HCV infection on daily activities, work ability and employment, particularly in the context of extended and long-term of interferon treatment. Several potential causes have been proposed including both direct invasion of the brain and secondary inflammatory immune responses provoked by HCV virus in the central nervous system [9, 10]. Silybin is a component of silymarin, a mixture of flavolignans extracted from the milk thistle. The *Silybum marianum* and its extract showed hepatoprotective [11], antioxidant [12], anticancer [13], anti-inflammatory [14], antifibrotic [11], immune modulator [14], sedative and anti-depressant properties [15, 16]. Therefore, increasing the antioxidant capacity of neurons it may provide a potential strategy to protect neurons [16]. Silybin has also known to be able to elevate some neurotransmitters concentration in brain [8, 16, 17]. Silybin may be useful in diseases known to be aggravated by reactive oxygen species and in the development of novel treatment for neurodegenerative disorders such as Alzheimer’s disease [16–18]. A possible involvement of source of oxidative stress in the development of anxiety, depression and suicidal behavior must be considered [19]. Prevention of mitochondria- derived ROS production by consumption of specific nutrients, such as fish oil, vitamin B12, folic acid, L-carnitine and its derivatives may help to prevent the onset of mood disorders in vulnerable subject such as HCV patients [20–22].

The aim of this study was to determine the efficacy of Silybin-vitamin E-phospholipids complex in reducing depressive and anxiety symptoms and improving work ability as measured by the Work Ability Index (WAI) in subjects with chronic hepatitis C treated with Peg-IFN-α and RBV.

## Methods

The study population was designed as a prospective, randomized, placebo controlled double-blind clinical trial. Eligible patients were male and female adults who had HCV RNA detectable in serum by polymerase chain reaction (PCR), had a liver biopsy within 12 months before study entry consistent with chronic hepatitis and had persistently elevated serum values of alanine aminotrasferase during the 30 day periods preceding the initiation of the test drug. Previous unsuccessful therapy attempts with less effective treatments were not an exclusion criterion. In addition, haemoglobin ≥12 mg/dl for woman and ≥13 mg/dl for man, white blood count (WBC) ≤11 × 10^9^/L, neutrophil count >1.5 × 10^9^ L, platelets ≤9 × 10^9^/L and bilirubine, albumin and creatinine in normal limits were required for enrollment. Ineligible patients were those who had decompensated cirrhosis, serum α-fetoprotein concentration of more than 50 mg/ml, HIV infection, cancer, severe jaundice, pulmonary and renal chronic disease, pre-existing psychiatry disease, autoimmune- type disease, poorly controlled diabetes, renal chronic disease seizure disorders, cardiovascular disease, haemoglobinopathies or if they were unable to use contraception. Other reason for exclusion had been serious medical disorders that would preclude treatment with interferon, interferon intolerance, active use of illicit drugs, active alcohol abuse, a suicide attempt or hospitalization for depression within the past 5 years. Standard demographic, clinical, medication, laboratory and radiological data were obtained.

The study was conducted at the Department of Medicine, University of Catania (Italy), between February 2010 and July 2013. This study was approved by Cannizzaro Hospital Ethics Committee. All sensitive data were collected and protected in respect of present privacy statements.

**Fig. 1:**
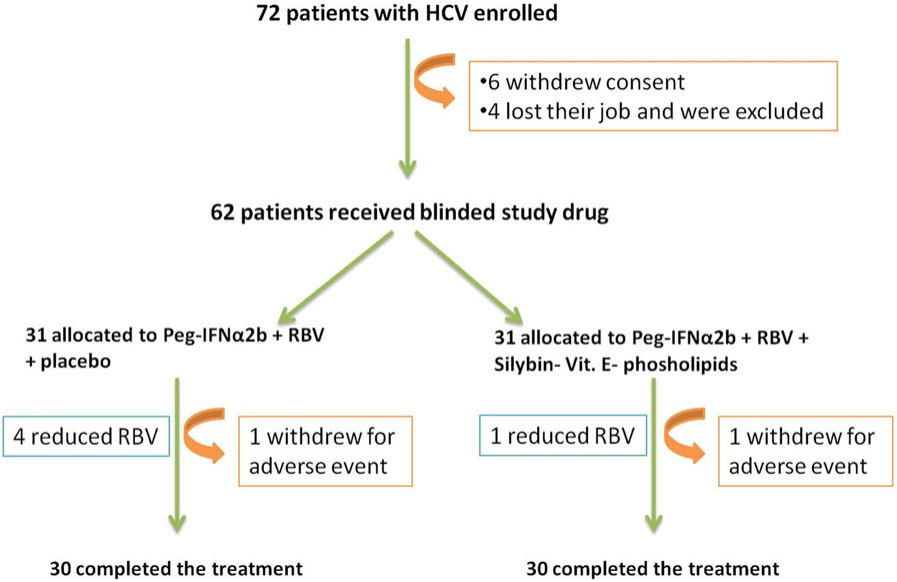
Trial profile of Peg-IFNα2b plus RBV plus Silybin- Vitamin E- phospholipids treatment

Sixty-two patients have been enrolled (36 males, 26 females) (Tables 1 and 2). In fact, for sample size determination (power 90%, α = 0.05 and dropout rate = 20%) was assumed and yielded a sample size of 62 patients in total (10, 11). The patients received Peg-IFN-α2b plus RBV (group A; *n* = 31) and placebo or Peg-IFN-α2b plus RBV plus Silybin 94 mg - vitamin E 30 mg - phospholipids 194 mg in pills (group B; *n* = 31) for a 12-month period (Fig. 1). Patients were randomized into two groups (Silybin - vitamin E- phospholipids complex versus placebo [23]) using permuted-block randomization with an allocation ratio of 1:1 and a block size of 4. Randomization was performed by an independent statistician. According to the sequence of their inclusion and patients received respective study products, random numbers were assigned to patients. Both clinical investigators and patients were blind to the product given: all drugs complex Silybin phosphatidylcholine and placebo were identically in appearance. Investigators and patients were informed of the selected agent just at the end of Peg interferon and Ribavirin treatment. Dosing instruction was provided with each patient pack. All trial medication was instructed to be taken as prescribed. Patients were considered compliant if the number of returned pills was between 80 and 120% of the planned treatment regimen. Any concomitant drugs were administered for the duration of the trial at the lowest possible therapeutic dose and, as much as possible, the therapeutic doses were not changed. Peg-IFN-α2b (1.5 mg/kg per week) plus RBV and placebo were administered to subjects in Group A. The dose of RBV was 800 mg for body weight less than 60 kg, 1000 mg between 60 and 75 kg, and 1200 mg more than 75 kg. Subjects in Group B received Peg-IFN-α2b and RBV plus Silybin - vitamin E - phospholipids complex administered three times a day per os. Subjects were evaluated before starting therapy, after 6 and 12 months. A follow-up was carried out 6 months after the end of the treatment. Study recruitment was performed in observation and respect of Helsinki Declaration [24]. All patients gave their written informed consent for the study participation and for each invasive procedure (hepatic biopsy) they underwent (Fig. 1).

**Table 1:** Patients characteristics at liver biopsy

Parameter	Group A *n* = 31 (Peg-IFNα + RBV+ placebo)	Group B *n* = 31 (Peg-IFNα + RBV + Silybin, Vit E, phospholipids)	*p*-value
Male	18	17	NS
Female	13	14	NS
Route of transmission of HCV (No of patients)			
Blood transfusion	16	12	NS
Intravenous drug abuse	3	5	NS
Occupational	1	3	NS
Unknown	11	11	NS
HCV genotype			
1a	2	2	NS
1b	23	23	NS
2a	3	3	NS
3a	3	3	NS
Blue collars (manual laborers)	11	11	NS
White collar (non manual/office laborers)	20	20	NS

*NS* not significant

**Table 2:** Patients characteristics at liver biopsy. Values are expressed as Mean ± SD

Parameter	Group A *n* = 31 (Peg-IFNα + RBV + placebo)	Group B *n* = 31 (Peg-IFNα + RBV + Silybin, Vit E, phospholipids)	*p*-value
Mean age (years)	45.8 ± 3.9	47.2 ± 3.7	N.S.
HCV exposure time (years)	5.87 ± 4.9	5.96 ± 4.2	N.S
BMI (kg/m^2^)	27.8 ± 3.1	27.4 ± 3.6	N.S.
Plasma glucose (mmol/l) (normal 3.9–6.4)	5.6 ± 0.78	5.9 ± 0.70	N.S.
AST (IU/l) (normal 15–50)	173.2 ± 37.8	178.4 ± 37.4	N.S.
ALT (IU/l) (normal 15–50)	187.2 ± 37.2	184.1 ± 38.2	N.S.
Viremia (10^6^ IU/ml)	5.21 ± 2.24	5.20 ± 2.44	N.S.
HAI	10.7 ± 3.2	10.9 ± 3.4	N.S.

### Serum analysis

All patients underwent a virological assay for HBsAg (Hepatitis B surface Antigen). Anti-HCV antibodies were determined by ELISA (Ortho Diagnostic Systems, Raritan, NJ, USA). HCV-RNA (Hepatitis C Virus RNA) levels were detected by polymerase chain reaction (PCR) of HCV-RNA 5″UTR using COBAS AmpliPrep/COBAS TaqMan (Roche Diagnostics Systems, Branchburg, N.J). HCV viral genotypes were determined by restriction analysis of HCV-RNA 5″UTR [25, 26]. Aspartate Aminotransferase (AST) and Alanine Aminotransferase (ALT), gamma Glutamil Tranferase (γGT), total, conjugated and unconjugated bilirubin, serum proteins analysis were performed. All liver function tests, hematochemical measurements, and virological analysis have been executed in the laboratory of our hospital with automated and standardized methods.

### Histological grading assessment

Patients underwent ultrasound-assisted percutaneous biopsy: tissue specimens were obtained with Menghini modified needles (Automatic Aspiration Needle for Liver Biopsy, ACR 16G, 11 cm, manufactured by Sterylab Srl, Milan-Italy). A biopsy was considered adequate for evaluation if the specimen was >1.5 cm long and contained a minimum of 6 portal tracts. Knodell and Ishak Histological activity index (HAI) score was used to assess the histological grading of the disease [27].

### Beck depression inventory (BDI)

The B.D.I is a self-assessment instrument consisting of 21 items, which asses the severity of symptoms and attitudes related to depression. The sum of the scores obtained in each item results in a total score ranging from 0 to 63. Accordingly, BDI score was calculated. Index score of ≤9 is considered to be within normal range, a score of 10–15 shows minimal depressive symptomatology, a score of 16–31 points toward mild depression, a score of 32–47 is in favor of moderate depression and a score of >47 indicates severe depression [28].

### State-trait anxiety inventory (STAI)

The STAI is intended for self- reported measures of state and trait anxiety [29]. The state anxiety is an emotional state existing at a given moment in time and a particular level of intensity. It is characterized by subjective feelings of tension, apprehension, nervousness, and worry. The state anxiety form consists of 20 items expressed as statements that indicate how respondents feel “right now, at this moment”; responses are: 1) “not all”; 2) “somewhat”; 3) “moderately so”; and 4) “very much so”. The answers are transformed into weighted scores of 1–4, where 4 indicate the presence of high level of anxiety. However, 10 items are phrased so that high score reflex absence of anxiety. In those 10 items the weighted scores are reversed. Scores derived from the state anxiety form can vary from minimum of 20 (absence of anxiety) a maximum of 80 (high level of anxiety).

### Work ability index (WAI)

The Work Ability Index (WAI) is a validated tool for measuring self-assessed work ability and reveals how well a worker thinks she/he is able to perform her/his work. The instrument is used to measure work ability in health examinations and workplace surveys. Current work ability compared with the best lifetime evaluation is rated on an 11 points scale from 0 completely unable to work) to 10 (work ability at its best). Current physical and mental work ability is also rated on five-point Likert scales from 1 (very good) to 5 (very poor). The WAI also covers the “number of current diseases diagnosed by a physician” covering: injuries from accidents, musculoskeletal disease, cardiovascular disease, respiratory disease, mental disorder, metabolic disease, neurological or sensory disease, or other severe disease. The WAI also consisted of a scale for the following symptoms: tiredness, nervousness, concentration problems, headache, palpitation, vertigo, nausea, chest pain, stomach ache and insomnia. These items are scored on Likert Scales with five response alternatives from 0 (never) to 4 (the entire item). Symptom score range was from 0 (no symptoms) to 40 (maximum). Completion of the questionnaire results in score which lies between 7 and 49. WAI total score of 7–27means poor work ability work capacity 28–36 good, 37–43 very good, excellent 44–49 [30–32].

### Efficacy and safety assessment

The primary efficacy measure were changes in anxiety, depression and work ability before of treatment and after 1 month, 6 months, 12 months and at follow up. Moreover, the virological response in subject treated with Peg IFNα + Ribavirin + Silybin versus IFNα + Ribavirin plus placebo had been evaluated.

We performed an intention-to-treat (ITT) efficacy analysis. “Sustained virological responders” (SVR) were patients with not identifiable serum HCV RNA at the end of the study. We considered the “relapse” as undetectable HCV-RNA levels at the end of treatment but detectable levels during the follow-up period. Reasons for discontinuation of the treatment were severe adverse events and absence of compliance.

### Statistical analysis

Results are expressed as means ± standard deviations. Quantitative data were compared by paired or unpaired Students *t*-test or Mann–Whitney test; the χ-square was used for analysis of qualitative data. All results shown in this manuscript were analyzed in the intention-to-treat population. *P* values < 0.05 were considered statistically significant. All statistical analysis was performed using SPSS 15.0 (Chicago, IL).

## Results

Demographics characteristics were similar between the two groups at baseline (Tables 1 and 2). The most frequent viral genotype was 1b. At enrollment no significative differences were observed in biohumoral tests in characteristic at liver biopsy, in work ability index, in STAI and B.D.I.

### Effect of PEG-IFNα, RBV and Placebo

In group treated with PEG-IFNα + RBV+ Placebo (Group A) there was a significant decrease in AST (*p* < 0.001) and ALT (*p* < 0.002) after 6 months; whereas after 12 months and at follow-up in AST and in ALT (*p* < 0.001). Viremia was significantly reduced after 6 months (*P* < 0.05), 12 months (*P* < 0.001), and at follow up (*P* < 0.001) (Table 3). HAI score decreased after 12 months (*P* < 0.001). Work ability index was decreased after 1 month (*p* < 0.01). STAI score was increased after 1 month (*p* < 0.01), after 6 months (*p* < 0.01), after 12 months (*p* < 0.01), and at follow-up (*p* < 0.01). B.D.I. was increased after 1 month (*p* < 0.01), after 6 months (*p* < 0.01), after 12 months (*p* < 0.01) and at follow-up (*p* < 0.01) (Tables 3 and 4).

**Table 3:** Characteristics of subjects at baseline, after 12 months, and at follow-up. Values are expressed as Mean (SD)

Group A Peg-IFN α + RBV + placebo (*n* = 31)							
	Before treatment	After 6 months	*P*-value	After 12 months	*P*-value	Follow-up	*P*-value1
AST (IU/l)	173.2 ± 37.8	94.1 ± 37.2	<0.001	65.4 ± 21.8	<0.001	63.2 ± 22.9	<0.001
ALT (IU/l)	187.2 ± 37.2	156.2 ± 38.7	<0.001	70.4 ± 15.2	<0.001	76.2 ± 15.8	<0.001
Bilirubin (mmol/l)	10.6 ± 7.9	10.4 ± 6.7	NS	10.2 ± 6.6	NS	10.4 ± 6.7	NS
Albumin (g/dl)	4.1 ± 0.8	4.2 ± 0.7	NS	4.0 ± 0.8	NS	4.2 ± 0.6	NS
Viremia (10^6^ IU/ml)	5.21 ± 2.24	3.8 ± 2.1	<0.05	2.67 ± 1.9	<0.001	2.8 ± 1.8	<0.001
HAI	10.7 ± 3.2	-	-	7.9 ± 2.4	<0.001	-	-
Group B Peg IFN α + RBV + Silybin, Vit E, phospholipids (*n* = 31)							
	Before treatment	After 6 months	*P*-value	After 12 months	*P*-value	Follow-up	*P*-value
AST (IU/l)	178.4 ± 37.4	87.7 ± 31.4	<0.001	47.2 ± 16.8	<0.001	47.8 ± 18.2	<0.001
ALT (IU/l)	184.1 ± 38.2	78.8 ± 38.7	<0.001	39.4 ± 13.8	<0.001	41.8 ± 19.8	<0.001
Bilirubin (mmol/l)	10.2 ± 7.1	10.3 ± 6.9	NS	10.2 ± 3.1	NS	10.2 ± 4.9	NS
Albumin (g/dl)	4.1 ± 0.3	4.2 ± 0.6	NS	4.2 ± 0.8	NS	4.2 ± 0.6	NS
Viremia (10^6^ IU/ml)	5.20 ± 2.44	2.67 ± 1.9	<0.001	1.9 ± 1.7	<0.001	1.97 ± 1.8	<0.001
HAI	10.9 ± 3.4	-	-	7.9 ± 2.1	<0.001	-	-

**Table 4:** Stresses scores in the study groups. Values are expressed as Mean (SD)

	Before treatment	After 1 month	*P* value	After 6 months	*P* value	After 12 months	*P* value	Follow-up	*P* value
Group A Peg-IFN α + RBV + placebo (*n* = 31)									
Work ability index	36.9 ± 5.2	21.4 ± 5.3	<0.01	37.8 ± 5.9	NS	38.7 ± 5.8	NS	39.8 ± 6.7	<0.001
STAI	50.1 ± 7.6	59.2 ± 7.4	<0.01	60.4 ± 7.7	<0.001	58.2 ± 7.4	<0.001	61.4 ± 7.2	<0.001
BDI	30.8 ± 6.9	44.6 ± 6.7	<0.01	46.7 ± 6.8	<0.001	40.9 ± 6.9	<0.001	40.4 ± 6.2	<0.001
Group B Peg IFN α + RBV + Silybin, Vit E, phospholipids (*n* = 31)									
Work ability index	35.4 ± 5.9	30.2 ± 6.2	<0.001	38.4 ± 6.8	NS	40.7 ± 6.1	<0.05	39.8 ± 6.4	<0.05
STAI	50.8 ± 7.9	59.8 ± 7.1	<0.001	50.4 ± 7.2	NS	47.4 ± 7.8	NS	50.6 ± 7.0	NS
BDI	30.7 ± 7.1	48.7 ± 7.8	<0.001	34.6 ± 7.1	NS	30.1 ± 7.2	NS	30.4 ± 6.8	NS

### Effect of Silybin, vitamin E and phospholipids complex

In the group B treated with Peg-IFNα2b and RBV plus Silybin-vitamin E-phospholipids complex we observed a significant decrease in AST (*P* < 0.001), ALT (*P* < 0.001), and viremia (*P* < 0.001) after 6, 12 months, and at follow up. A significant decrease in HAI score (*P* < 0.001) was observed after 12 months. Work Ability Index was decreased after 1 month (*p* < 0.01) and increased after 12 months (*p* < 0.05) and at follow-up (*p* < 0.05). STAI score and B.D.I. were increased after 1 month (*p* < 0.01) (Tables 3 and 4).

### Comparison between Group A and B

The comparison between group A and group B showed a significant difference after 6 months in ALT (*P* < 0.001), and viremia (*P* < 0.05), after 12 months in ALT (*P* < 0.001), and AST (*P* < 0.001). At follow up, we observed a significant difference in AST (*P* < 0.05), and ALT (*P* < 0.001). In the comparison between group treated with Peg IFN plus Ribavirin plus Silybin complex and Peg IFN plus Ribavirin alone we observed after 6 months significant differences in AST (90.7 IU/L *p* < 0.001) in ALT (105.3 IU/L *p* < 0.001) and viremia (2.53 IU/ml *p* < 0.001), after 12 months in AST (131.2 IU/L *p* < 0.001) in ALT (144.7 IU/L *p* < 0.001) in viremia (3.1 IU *p* < 0.001) and at follow-up in AST (130 IU/L *p* < 0.001) in ALT (142.3 IU/L *P* < 0.001) in viremia (3.23 IU/ml *p* < 0.001) in HAI (3.0 *p* < 0.001). In the comparison between group treated with Peg IFN plus ribavirin plus Silybin and Peg IFN plus ribavirin alone we observed in WAI a decreased at 1 month (5.2 vs. 15.5 *p* < 0.01) and increased after 6 months (3.0 vs. 0.9 *p* < 0.01), after 12 months (5.3 vs. 1.8 *p* < 0.01), and at follow up (4.4 vs. 2.9 *p* < 0.05). We also observed increased in STAI after 1 month (9.0 vs. 9.1 NS), after 6 months (−0.4 vs. 10.4 *p* < 0.01), after 12 months (−3.4 vs. 8.1 *p* < 0.01), and at follow-up (−0.2 vs. 11.3 *p* < 0.01). Increase in B.D.I after 1 month (18.0 vs. 13.8 *p* < 0.01) and decrease after 6 months (3.9 v. 15.9 *p* < 0.01), after 12 months (0.6 vs. 10.1 *p* < 0.01), at follow up (0.3 vs. 11.6 *p* < 0.01) (Table 4).

### Effects of Peg IFNα + Ribavirin with or without Silybin on psychological outcomes and work ability

In the Group A treated with Peg IFNα and Ribavirin alone, we observed a significant decrease of WAI (*p* < 0.01) after 1, 6 and 12 months; a significant increase of absenteeism (*p* < 0.001) after 6 and 12 months and at follow-up a decrease of anxiety (*p* < 0.05) at 6 months and increase of depression (*p* < 0.01) at 1, 6, 12 and at follow up. In the Group B treated with Peg IFNα plus Ribavirin and Silybin complex, we observed a significant improve (*p* < 0.05) of WAI at 12 months and at follow-up; a significant decrease of absenteeism (*p* < 0.05) at 12 months and at follow-up; a significant reduction of depression at 12 month and at follow-up, and a reduction of anxiety at 6, 12 months and at follow-up. In comparison between Group A with Group B, we observed in Silybin treated group an improvement in WAI at 12 month and follow-up; a reduction of absenteeism at 12 months and at follow-up; a reduction of depression at 12 month and at follow-up; a reduction of anxiety at 6, 12 months and at follow-up (Tables 5 and 6).

**Table 5:** Stress grades: Group IFN and Ribavirin alone

	Before	After 1 month	After 6 months	After 12 months	Follow-up
Work ability Index					
*Excellent*	3	-	-	1	2
*Very good*	4	2	1	1	6
*Good*	21	20	21	22	20
*Bad*	3	9	9	7	3
Absenteeism					
*1-7 days*	28	26	24	24	20
*<4 weeks*	3	5	1	3	11
*<3 months*	-	-	6	4	-
*>3 months*	-	-	-	-	-
*>6 months*	-	-	-	-	-
Depression					
*Normal*	8	-	-	2	4
*Minimal*	6	8	9	11	10
*Mild*	3	8	7	5	7
*Moderate*	-	15	15	13	10
*Severe*	-	-	-	-	-
Anxiety					
*Absence*	5	1	3	5	7
*Mild*	14	15	8	11	10
*Moderate*	12	10	18	15	14
*High*	-	5	2	-	-

**Table 6:** Stress grades: Group supplemented with Silybin

	Before	After 1 month	After 6 months	After 12 months	Follow-up
Work ability Index					
*Excellent*	3	-	-	3	3
*Very good*	3	3	3	3	8
*Good*	21	22	23	25	20
*Bad*	4	6	5	-	-
Absenteeism					
*1-7 days*	27	26	25	20	21
*<4 weeks*	4	5	6	11	10
*<3 months*	-	-	-	-	-
*>3 months*	-	-	-	-	-
*>6 months*	-	-	-	-	-
Depression					
*Normal*	4	1	4	7	5
*Minimal*	8	8	11	14	15
*Mild*	4	4	4	-	3
*Moderate*	15	18	12	10	8
*Severe*	-	-	-	-	-
Anxiety					
*Absence*	2	1	4	7	6
*Mild*	15	18	17	14	15
*Moderate*	14	12	10	10	10
*High*	-	-	-	-	-

### Adverse effects

In our study population we observed no serious adverse events (WHO grade 3 or 4) have been observed in both groups. Adverse effects included hypercholesterolemia, musculoskeletal pain, myalgia, hypertrigliceridemia, hyperglycemia, irritability and weight loss as described in Table 7.

**Table 7:** Adverse events observed in the study population

	Group A (*n* = 31) (Peg-IFNα + RBV + placebo)	Group B (*n* = 31) (Peg-IFNα + RBV + Silybin, Vit E, phospholipids)	*P* Value
Psychological disorders	14%	12%	NS
Hypercholesterolemia	16%	32%	*P* < 0.01
Fatigue	48%	44%	NS
Headache	41%	42%	NS
Musculoskeletal pain	54%	35%	*P* < 0.01
Myalgia	58%	32%	*P* < 0.01
Hypertriglyceridemia	44%	30%	*P* < 0.01
Nausea	31%	24%	*P* < 0.05
Anorexia	6%	8%	NS
Irritability	30%	22%	*P* < 0.05
Hyperglycemia	13%	8%	*P* < 0.05
Weight loss	12%	7%	*P* < 0.05
Decrease of hemoglobin values at the end of treatment	from 13.6 g/dL (range 11.4–14.4) to 11.6 g/dL (range 10.4–14.2 g/dL)	from 13.6 g/dL (range 11.6–15.9 g/dL) to 10.6 (range 10.4–12.8 g/dL)	NS

## Discussion

Both HCV and IFN treatment often increase the stresses and burdens to patients, their families and society [33]. Chronic disease can impact psychological adjustment and social adaptation, gradually interfering with daily activities and decreasing people’s ability to function in society [34]. Work ability is a multidimensional phenomenon and is dependent on both mental and somatic health status as well as on social skills, on level of education, on motivation, on work demands, on work environment and on organization of the work. The HCV infection and related treatment with Peg-IFN plus RBV impairs work performance among office workers, which may lead to a substantial loss to work productivity. Many HCV patients report feelings of stigmatization and social isolation from their friends [35, 36]. Depressive symptoms prior to initiating antiviral treatment for HCV are associated with greater likelihood of developing major depressive disorder during treatment. It has been suggested that depression evaluation such as BDI, tend to overestimate depressive symptoms because the somatic symptoms (loss of energy, sleep changes, irritability, concentration difficulty, appetite changes, loss of libido, tiredness and fatigue) are also common symptoms of physical illness [37].

In our study we observed in HCV patients treated with Peg-IFN plus RBV alone an increase of depression score and anxiety associated with a decrease of work ability index after 1 month of treatment. The inflammatory and proinflammatory cytokines with active participation of genetic and environmental factors may trigger the depression development and the consequent absenteeism [7]. Slightly more than 50% of patients reduced their work schedule at least one time, although 71% of patients returned to former work schedules. Working HCV patients treated with Peg-IFN plus RBV reduced their weekly hours worked by nearly 20% at 6 months following treatment. The absenteeism was associated with various phases of therapy more physical, mental and psychological symptoms. Although depression is often not supported by biohumoral, virological and histologic data, it may develop in anxiety and pessimism in patients treated with IFN and RBV [38, 39]. These symptoms are evident during the first months, but when Silybin is associated with IFN and RBV these symptoms are reduced. When comparing the two study groups, we observed increased work ability and reduced depression and anxiety in patients treated with Peg–IFN and RBV plus Silybin-vitamin E-phospholipids complex. The reduction in anxiety symptoms associated with Silybin-vitamin E-phospholipids complex provides the first evidence that Silybin may have potential anxiolytic benefits. Earlier studies have reported that Silybin has strong antioxidant and anti-inflammatory effect which are beneficial in cognitive impairment. In HCV patients, including non responders to a previous treatment with IFN + RBV, Silybin treatment showed a decrease in symptoms and an increase of various aspect of quality of life [39, 40].

In the patients that used Silybin the univariate and multivariate analysis, documented a lower incidence of depression, anxiety and irritability, and a better quality of life in patients Silybin treated than those who did not. Our study showed that HCV patients treated with Silybin-vitamin E-phospholipids complex supplementation benefited clinically in patience, depression, anxiety, and in the most common outcomes of stress interferon-induced. Moreover alteration of behavioral, a reduction of anxiety and the diffuse state of distress with symptoms of hyperarousal and pointless worries had been documented [41]. Anxious subjects show a cognitive bias in that they pay increased attention to threat-related cues, and tend to interpret emotionally ambiguous stimuli in a threatening manner. These cognitive biases are thought to underlie avoidance behavior, e.g. avoidance of social contact, as a cardinal symptom of anxiety disorders [42], which suggests a change in the way patients perceive and deal with stressors and work demands. Depression is a complex heterogeneous disorder with a wide spectrum of anomalies including depressed mood, anhedonia, sleep disorders, fatigue, loss of self-esteem, negative thinking and suicide thoughts [43]. In the comparison with two groups we observed significant differences in WAI, STAI, and B.D.I in subjects supplemented with Silybin (Table 7). Administration of Peg Interferon α and Ribavirin may reach eradication rates of 45 and 52% in patients with genotype 1. Patients, who have genotype 2 and 3, usually have a shorter duration of treatment and a better prognosis. Nevertheless, in our study no one of these patients had early negative serum levels of HCV RNA.

A fairly synchronous occurrence of both decreased severity of depressive symptoms and improvement in work ability has also been found in longitudinal cohort studies, although improvements in symptoms also have been reported to occur more rapidly than improvements in the area of work [44]. The study limitations include the small number of patients. Another limitation is that comparisons were made between a real disease that these patients were suffering from, and perceived rather than real losses. However, perceived stigmatization by physicians and a sense of abandonment reflect the need for educational efforts and for the family whom they live with. The supplementation with Silybin-vitamin E-phospholipids complex increased the sustained virological response in patients treated with interferon and ribavirin [11]. In fact, in sustained virological responders, work ability is increased compared with non responders. In HCV patients, an improvement in work capability could reflect higher work productivity as well as better well being and quality of life. However, there are several competing interpretations because of which no firm conclusion can be made yet. First, it is hypothesized that the effects on the realization and sustenance of work ability emerge only after a follow-up longer: at least 3 years [45]. Second, the selection of the patients’ population with low level of personality disorders and realized sickness incapacity at baseline may be more suitable for short-term therapy, and thus influence the effects. Moreover, the participants were recruited from a single centre. This may have influenced the socio-demographic characteristics of the sample and results cannot be considered as representative of general population.

Direct acting antiviral agents have revolutionized HCV therapy. Although interferon free therapy may be a preferred option, some patients may still require interferon based regimen to ensure efficacy [46]. In fact IFN remains a backbone of many new regimens, the addition of these novel agents are likely to result in increased tolerability and efficacy when compared to the current treatment [47, 48]. Modern studies of the induction and biological activity of IFN in HCV infection have not only promoted our understanding of the pathogenesis of the liver disease worldwide but also made important contribution to uncover the mysteries of virus host interaction [49, 50]. The interferon system provides a powerful antiviral response, which inhibits the replication of several viruses, including HCV stimulates apoptosis of infected cells, modulates the immune system, and the consequence spectrum of psychiatric disorders [51, 52]. The complex interplay between the host and both HCV and IFN-α plus ribavirin is a fascinating one. The direct effects of HCV on Central Nervous System and Interferon treatment are a strong influence on psychological distress and on work and consequently on daily life. The conjugation of complex Silybin + phosphatidylcholine and Vitamin E induced a greater solubility of Silybin.

## Conclusion

The complex Silybin + phosphatidylcholine (Silybin phytosome was formulated with the addition of Vitamin E) (Lorenzin Spa Italy), it not only increase the response to Peg-IFN and Ribavirin in patients but also reduce depression, anxiety and improve work ability in HCV patients [23].﻿

Further studies are needed to deeply understand the factors related to employment, to maintain and return to work (including what related to sick leave, job loss, and length work changes), employer satisfaction, career changes, work ability and perceived job strain, the related quality of life, and both physical and psychological disablement [53].
